# Omentum Mimicking as a Vaginal Prolapse in a Delayed Vaginal Cuff Dehiscence

**DOI:** 10.7759/cureus.50647

**Published:** 2023-12-17

**Authors:** Kanika Gupta, Vivek Mangla, Sanjeev Arora, Gautam Anand, Shubham Bidhuri

**Affiliations:** 1 Gynecologic Oncology, Max Healthcare, New Delhi, IND; 2 Gastrointestinal Surgery, Max Healthcare, New Delhi, IND; 3 Surgical Oncology, Max Super Specialty Hospital, Vaishali, Ghaziabad, IND

**Keywords:** minimally invasive procedure, vaginal hysterectomy, total laparoscopic hysterectomy, omental prolapse, vaginal cuff dehiscence

## Abstract

A rare consequence of hysterectomy is vaginal vault dehiscence, which commonly occurs five to seven weeks after the procedure. Its frequency ranges from 0% to 7.5%. The incidence of delayed dehiscence is rare. The small bowel is the organ that prolapses most frequently, but other organs and multi-organ prolapses have also been documented. Due to potential catastrophes such as intestinal ischemia, blockage, and perforation, transvaginal protrusion of abdominal viscera is an emergency. A laparoscopic approach facilitates a thorough evaluation of the abdominal contents and provides assistance in challenging circumstances where the contents are not reducible.

## Introduction

Patients with a history of hysterectomy can experience vaginal cuff dehiscence. Abdominal contents may protrude into the vagina through the vaginal cuff. Its frequency ranges from 0 to 7.5% [1]. Typically, it occurs five to seven weeks following surgery [2]. The incidence of delayed dehiscence is rare. The small bowel is the organ that prolapses most frequently, but other organs and multi-organ prolapses have also been documented [3]. To avoid further complication, abdominal organ prolapse requires immediate medical attention [4]. We describe an instance of delayed vaginal vault dehiscence with omental prolapse treated with a laparoscopic approach six months after the total laparoscopic hysterectomy.

## Case presentation

**Figure 1:**
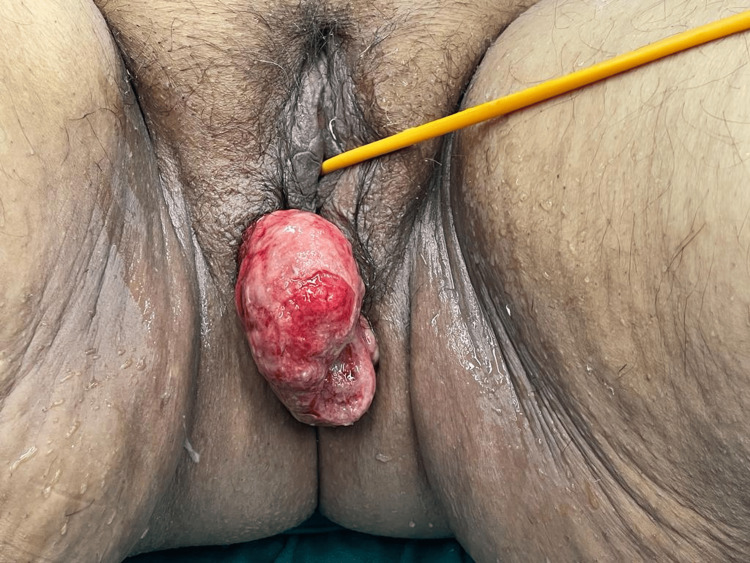
External pelvic examination showing a necrosed omental herniating through the vagina. Content was irreducible.

A 53-year-old lady without any comorbidity underwent total laparoscopic hysterectomy for uterine fibroids six months back. She complained of pain in the lower abdomen with something coming out per vaginum for the last five days. On examination, her vitals were stable. On per abdomen examination, the abdomen was soft, non-tender, with no guarding or rigidity present. On perineal examination, there was omental herniation through a defect in the vaginal vault. The prolapsed content was dusky and necrosed, and it was irreducible (Figure 1).

**Figure 2:**
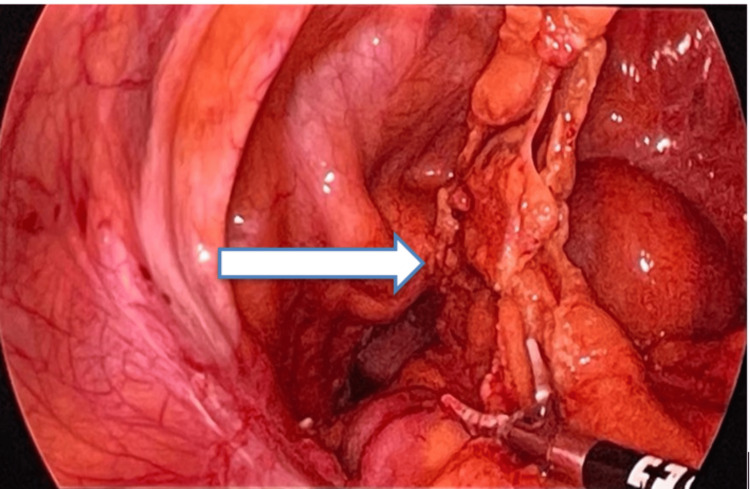
Intraoperatively, on laparoscopy, omentum prolapsing through the dehisced vaginal cuff (marked by arrow).

The patient was immediately planned for examination under anesthesia. Peroperative findings confirmed the herniating mass as necrosed omentum through the dehisced vaginal cuff. She underwent diagnostic laparoscopy with excision of the prolapsed omentum. Laparoscopic adhesiolysis was performed, and the margins of the vaginal vault were identified (Figure 2).

**Figure 3:**
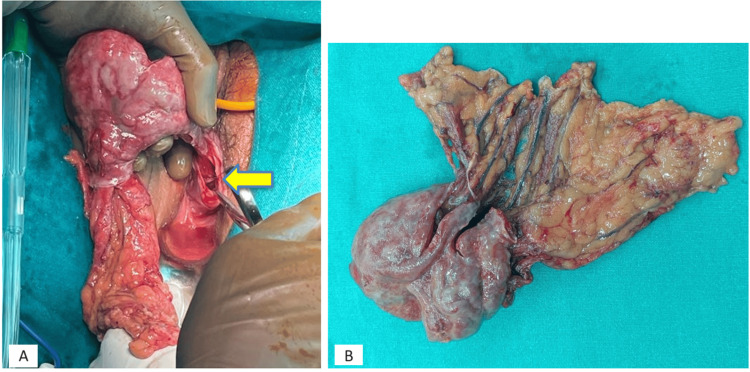
(A) Specimen delivered per vaginally after ligating the omental stalk laparoscopically (yellow arrow showing dehisced vaginal vault margin). (B) Final specimen showing necrosed and dusky prolapsed part with the normal omentum proximally.

After excision of the prolapsed omentum, the specimen was retrieved transvaginally. The vaginal vault defect closure was done using Prolene 1.0 sutures after refining the margins (Figure 3).

The patient tolerated the procedure well, and the postoperative period was uneventful. She was discharged on the third postoperative day. Now, in the routine follow-up, she is doing well.

## Discussion

A rare consequence of hysterectomy is vaginal vault dehiscence, which commonly occurs five to seven weeks after the procedure [2]. In our case, a rare incident of delayed dehiscence, the vault dehiscence with omentum prolapse, occurred six months after the surgery. In comparison to total abdominal hysterectomy (0.15%), total vaginal hysterectomy (0.08%), and laparoscopic-assisted vaginal hysterectomy (0.28%), vaginal cuff dehiscence is projected to occur at a rate of 0.4%. It is more frequent after total laparoscopic hysterectomy (1.36%) [5]. Risk factors for vault dehiscence include old age, diabetes, postmenopausal status, smoking, steroids, and poor technique of vault closure [6]. The patient might also experience genital pain, pelvic discomfort, and vaginal discharge. Although the evisceration of the colon, omentum, adnexa, urinary bladder, appendix, and even numerous organs has been recorded in the literature, the small bowel is the most often eviscerated organ [3]. Due to potential catastrophes such as intestinal ischemia, blockage, and perforation, transvaginal protrusion of abdominal viscera is an emergency. To prevent further injury, it is advisable to wrap the contents with a moist mop, and the patient must be placed in the supine position. The treatment of vaginal vault dehiscence with abdominal content prolapse is not standardized.

The three kinds of procedures discussed in the literature are transvaginal, transabdominal open, and transabdominal laparoscopic [7]. The choice of procedure depends on the evisceration's content, the contents' reducibility and ischemia, the presence of an intra-abdominal collection, the level of expertise on hand, and the stability of the patient. The least invasive method appears to be transvaginal, suitable when the contents are small, easily reducible, and there are no signs of ischemia or intra-abdominal collection. However, the biggest drawback of the transvaginal approach is its limited ability to view the abdominal contents. The transabdominal approach is preferred when the contents are ischemic and irreducible, there are abdominal symptoms present, and there are intra-abdominal collections. The transabdominal open approach offers a clearer view of the abdominal contents and allows for easy bowel or omentum resection, but the main disadvantage is the morbidity of open surgery. Over the two techniques mentioned above, the laparoscopic transabdominal approach has the benefit of being less invasive and enabling visibility of the abdominal viscera [8].

To prevent sepsis and peritonitis, reduced contents must be thoroughly checked, and any ischemic bowel or omentum must be removed. After the contents have been reduced, it is crucial to properly close the vault to avoid another incident.

## Conclusions

Vaginal vault dehiscence following hysterectomy is rare, often occurring five to seven weeks after the operation. Delayed dehiscence is even rarer. Vault dehiscence can cause several abdominal organs to prolapse; this condition needs immediate treatment to avoid future consequences. The laparoscopic approach facilitates a thorough evaluation of the abdominal contents and provides assistance in challenging circumstances where the contents are irreducible. It offers the advantages of a minimally invasive procedure.
